# PREFERENCES FOR NEW MODELS OF SOCIAL CARE FOR OLDER PEOPLE IN ENGLAND

**DOI:** 10.1093/geroni/igad104.1797

**Published:** 2023-12-21

**Authors:** Magdalena Walbaum

**Affiliations:** London School of Economics and Political Science, London, England, United Kingdom

## Abstract

Ageing and increasing diversity of the population pose challenges for social care provision. England has implemented several initiatives to improve care quality and make it more personalised, informed, and connected. This study aimed to understand the preferences of older people, including those from diverse backgrounds, for different aspects and models of social care, and how they trade-off different features of care. The study included two workstreams with participants aged 50+: 1) A qualitative study that explored preferences across five themes: housing settings, community assets, technology use, provision of care, and control and dignity; 2) A quantitative survey that analysed variations in preferences by demographic, socioeconomic, and needs-related factors. A discrete choice experiment was also conducted to evaluate the strength of preferences for different attributes. Older people value their independence and control over their lives and prioritise access to community resources and social connections. Participants also emphasised the importance of building a positive relationship with their care provider, respecting their sexual identity and personal beliefs to receive good quality care. In this presentation, differences in preferences among demographic, socioeconomic, and ethnic groups will be highlighted, underscoring the need to ensure that care packages align with individual preferences, beliefs, and values and promote equitable access to care, and outcomes. This approach may ensure that older people from diverse backgrounds receive high-quality care that promotes their independence and dignity and produces equitable care outcomes.

